# Relationship between body mass index and the expression of hormone receptors or human epidermal growth factor receptor 2 with respect to breast cancer survival

**DOI:** 10.1186/s12885-015-1879-4

**Published:** 2015-11-06

**Authors:** Ye Won Jeon, Su Hwan Kang, Min Ho Park, Woosung Lim, Se Heun Cho, Young Jin Suh

**Affiliations:** 1 Department of Surgery, St. Vincent’s Hospital, College of Medicine, The Catholic University, 93 Joongboo-Daero Paldal-gu, Suwon, 442-723 Kyunggi-do Republic of Korea; 2 Department of Surgery, Yeungnam University College of Medicine, 170 Hyunchung-ro, Nam-gu, Deagu, 705-703 Republic of Korea; 3 Department of Surgery, Chonnam National University Medical School, 160 Baekseo-ro, Dong-gu, Gwangju, 501-746 Republic of Korea; 4 Department of Surgery, Ewha Womans University, Mokdong hospital, 1071, Anyangcheon-ro, Yangcheon-gu, Seoul, 158-710 Republic of Korea; 5 Department of Surgery, Dong-A University College of Medicine, 26, Daesingongwon-ro, Seo-gu, Busan, 602-715 Republic of Korea

**Keywords:** Breast neoplasms, Body mass index, Survival, Estrogen receptor, Progesterone receptor, Human epidermal growth factor receptor 2

## Abstract

**Background:**

The association between body mass index (BMI) at the time of breast cancer diagnosis and the prognosis of breast cancer patients remains controversial. Furthermore, the association between BMI and prognosis with respect to different breast cancer subtypes is not clearly defined.

**Methods:**

We analyzed data from 41,021 invasive breast cancer patients between January 1988 and February 2008 from the Korean Breast Cancer Registry (KBCR) database. Overall survival (OS) and breast cancer-specific survival (BCSS) were analyzed using the Kaplan-Meier method and Cox’s proportional hazard regression model among all patients and specific breast cancer subtypes with respect to BMI categories.

**Results:**

A U-shaped association between BMI and mortality was observed in the total cohort. Underweight and obese individuals exhibited worse OS (hazard ratio, 1.23 [95 % confidence interval {CI}, 1.05 to 1.44] and 1.29 [1.13 to 1.48], respectively) and BCSS (1.26 [1.03 to 1.54] and 1.21 [1.02 to 1.43], respectively) than normal-weight individuals. In the estrogen receptor (ER) and/or progesterone receptor (PR)+/human epidermal growth factor receptor 2 (HER2) - subgroup, obese individuals exhibited worse OS (1.48 [1.18 to 1.85]) and BCSS (1.31 [1.13 to 1.52]) than normal-weight individuals. Conversely, in the ER and PR-/HER2+ subgroup, underweight individuals exhibited worse OS (1.68 [1.12 to 2.47]) and BCSS (1.79 [1.11 to 2.90]) than normal-weight individuals.

**Conclusions:**

We observed a U-shaped relationship between BMI at diagnosis and poor OS and BCSS among all breast cancer patients. However, obesity in the ER and/or PR+/HER2- subgroup and underweight in the ER and PR-/HER2+ subgroup were poor prognostic factors. Therefore, BMI at diagnosis and breast cancer subtype should be considered simultaneously in various treatment decision processes and surveillance schedules.

## Background

The association between body mass index (BMI) at the time of breast cancer diagnosis and the prognosis of breast cancer patients remains controversial despite many studies, including single institution, multi-center, and population-based studies, meta-analyses, and randomized controlled trials [1–24]. In many studies, a high BMI at the time of breast cancer diagnosis has been identified as a negative prognostic factor [1–17]. However, several studies have suggested that a low BMI at the time of breast cancer diagnosis correlates with a negative prognosis in breast cancer patients [18–20]. Some investigators have reported a weak or no relationship between BMI and prognosis in breast cancer patients [21–24].

Previous studies have not adequately demonstrated an association between BMI at the time of breast cancer diagnosis and prognosis in breast cancer patients with respect to breast cancer subtypes. Recent advances in our understanding of breast cancer biology based on molecular techniques allow us to divide breast cancer into at least four subtypes [25, 26]. These breast cancer subtypes exhibit different prognoses according to the estrogen receptor (ER), progesterone receptor (PR) and human epidermal growth factor receptor 2 (HER2) expressions. Therefore, it is important to understand the association between BMI and prognosis in the different breast cancer subtypes.

Moreover, there are certain differences between Asian and Western regions with respect to the prevalence of obesity. Although the prevalence of obesity is lower in Asians, the health risks associated with obesity occur at a lower BMI in Asian populations [27–29]. Therefore, an analysis of a large population-based cohort is needed to understand the prognostic significance of obesity in Asian breast cancer patients.

The aim of this study was to investigate the prognostic significance of BMI at the time of breast cancer diagnosis in all breast cancer patients and in each breast cancer subtype by analyzing overall survival (OS) and breast cancer-specific survival (BCSS) using population-based data from the Korean Breast Cancer Registry (KBCR) database.

## Methods

### Korean breast cancer registry (KBCR)

The KBCR database is a web-based, prospectively maintained nationwide database managed by the Korean Breast Cancer Society (KBCS). One hundred and two institutions have voluntarily participated in this registry since 1997. Before inserting personal information along with various datasets, written informed consent should be mandatory from the patient. From the initial conception of KBCR database, principal investigators from every single institution have agreed on the principles and process of utilizing this database for research purposes. After 2000, an online registration program was implemented, and the database has been actively utilized for various research studies on breast cancer in Korea [18, 30]. Essential registry items include the patient’s unique Korean resident registration number, gender, age, the surgical method used, and cancer stage according to the seventh edition of American Joint Committee on Cancer classification [31]. Moreover, data on height, weight, biological status (such as ER, PR, HER2, p53, and Ki67 status), and adjuvant treatment (such as radiotherapy, chemotherapy, and hormonal therapy) are collected as optional items within the KBCR database. The Korean Central Cancer Registry provides mortality data only, and the KBCR does not include information on tumor recurrence.

According to the guidelines of utilizing KBCR database, this study was approved by the institutional Review Board (IRB) of St. Vincent’s Hospital, College of Medicine, The Catholic University, where the first author of this article is affiliated (VC14RISI0234).

### Patients and follow-up

In this study, we selected and assessed invasive breast cancer patients who underwent curative surgery between January 1988 and February 2008. To achieve a more accurate analysis, we excluded patients treated with neoadjuvant therapy and patients for whom essential registry data (gender, age, height, weight and cancer stage) and ER/PR status were not available. Patients with distant metastasis at the time of diagnosis were excluded, because distant metastasis is the worst prognostic factor compared with other prognostic factors (such as age, tumor size, histologic grade, lymph node status, adjuvant treatment, BMI, hormone receptor status and HER2 expression) and serves as confounding factor for survival analysis.

The data on the remaining 41,021 patients were included in the final analysis.

All patients were categorized into five subgroups according to the expression of ER, PR and HER2 as follows: (a) ER and/or PR+/HER2-; (b) ER and/or PR+/HER2+; (c) ER and PR-/HER2+; (d) ER and PR-/HER2-; and (e) unknown. All patients for whom ER/PR expression but not HER2 expression information was available were categorized into the unknown group.

Positive staining for ER or PR was defined as the positive staining of ≥10 % nuclei in ten high-power fields, and HER2 positivity was defined as 3+ immunohistochemical (IHC) staining or HER2 gene amplification by fluorescence in situ hybridization (FISH). Cases of 2+ HER2 by IHC without a FISH result were treated as HER2-negative.

Patient survival data, including the date and cause of death, were obtained from the Korean Central Cancer Registry, Ministry of Health and Welfare, Korea.

### Statistical analysis

BMI was calculated by dividing weight (kg) by height (m) squared. The BMI at diagnosis was categorized as normal BMI (18.5 - 24.9 kg/m^2^), underweight BMI (<18.5 kg/m^2^), overweight BMI (25.0 – 29.9 kg/m^2^) and obese BMI (≥30 kg/m^2^) according to the guidelines of the World Health Organization (WHO) [32].

The patient characteristics were compared with respect to BMI category (underweight, normal weight, overweight and obese) using the chi-square test. The chi-square test and analysis of variance (ANOVA) were used to determine differences in the clinicopathological features between groups. With respect to survival analyses, we explored OS and BCSS using data from the KBCR database. OS was defined as the time from the initial diagnosis of primary breast cancer to death from any cause. BCSS was defined as survival until death from breast cancer. Survival curves were estimated using the Kaplan-Meier method. Log-rank tests were performed for the comparison of survival curves. Multivariate analyses were conducted using Cox’s proportional-hazard regression models to study the effect of BMI at diagnosis on OS and BCSS. The parameters included in the multivariate analysis model were as follows: patient age; tumor size; histologic grade; lymph node status; operation method; adjuvant treatment; ER/PR status and HER2 expression. A *p* value of less than 0.05 was considered significant. All statistical analyses were performed using the SAS software for Windows (release 9.2; SAS Institute, Cary, NC, USA).

## Results

### Patient characteristics

For the 41,021 patients included in our analysis, the mean age at breast cancer diagnosis was 48 years (range, 18 to 93). The baseline characteristics are presented in Table 1, stratified according to BMI categories. The BMI categories revealed a significant association with known breast cancer prognostic factors. The median age at diagnosis for obese patients was significantly older than underweight patients (*p* < 0.001). Obese patients had larger tumors (*p* < 0.001), high frequencies of axillary lymph node metastasis (*p* < 0.001), histologically high-grade lesions (*p* = 0.003), and negative ER and PR (*p* = 0.002) and HER2 expression (*p* = 0.004) compared with underweight patients.

**Table 1:** General characteristics of KBCS breast cancer subjects, overall and by BMI categories

Characteristic	Overall	Underweight BMI	Normal BMI	Overweight BMI	Obese BMI	*p* value
		<18.5 kg/m^2^	18.5-24.9 kg/m^2^	25-29.9 kg/m^2^	≥30 kg/m^2^	
No. of subjects	41021	1387	27519	10483	1632	
Age (years)						
mean(SD)	48(10)	42(10)	47(10)	52(10)	53(11)	<0.0001
Median(Range)	47(18-93)	41(19-87)	46(18-93)	51(19-90)	53(22-87)	
≤ 35	3737(9.11)	390(28.12)	2793(10.15)	483(4.61)	71(4.35)	<0.0001
> 35	37284(90.89)	997(71.88)	24726(89.85)	10000(95.39)	1561(95.65)	
Tumor size (cm)						
0 - 2	21931(53.46)	827(59.63)	15331(55.71)	5083(48.49)	690(42.28)	<0.0001
> 2	19090(46.54)	560(40.37)	12188(44.29)	5400(51.51)	942(57.72)	
Axillary lymph node metastasis						
Negative	25205(61.44)	916(66.04)	17205(62.52)	6153(58.70)	931(57.05)	<0.0001
Positive	15816(38.56)	471(33.96)	10314(37.48)	4330(41.30)	701(42.95)	
Operation method						
Mastectomy	23362(56.95)	759(54.72)	15361(55.82)	6254(59.66)	988(60.54)	<0.0001
Conserving surgery	17659(43.05)	628(45.28)	12158(44.18)	4229(40.34)	644(39.46)	
ER/PR expression						
ER and/or PR Positive	28166(68.66)	970(69.94)	19065(69.28)	7040(67.16)	1091(66.85)	0.0002
ER and PR Negative	12855(31.34)	417(30.06)	8454(30.72)	3443(32.84)	541(33.15)	
HER 2 expression						
Negative	28530(69.55)	955(68.85)	18995(69.03)	7401(70.60)	1179(72.24)	0.0004
Positive	8005(19.51)	265(19.11)	5541(20.14)	1917(18.29)	282(17.28)	
Unknown	4486(10.94)	167(12.04)	2983(10.84)	1165(11.11)	171(10.48)	
Subtype						
ER and/or PR + and HER 2 -	21094(51.42)	706(50.90)	14179(51.52)	5363(51.16)	846(51.84)	<0.0001
ER and/or PR + and HER 2 +	4118(10.04)	144(10.38)	2909(10.57)	929(8.86)	136(8.33)	
ER and PR - and HER 2 +	3887(9.48)	121(8.72)	2632(9.56)	988(9.42)	146(8.95)	
ER and PR - and HER 2 - -	7436(18.13)	249(17.95)	4816(17.50)	2038(19.44)	333(20.40)	
Unknown	4486(10.94)	167(12.04)	2983(10.84)	1165(11.11)	171(10.48)	
Histologic grade						
Low (grade1 - 2)	20043(48.86)	690(49.75)	13495(49.04)	5050(48.17)	808(49.51)	0.0032
High (Grade 3)	15890(38.74)	498(35.90)	10560(38.37)	4186(39.93)	646(39.58)	
Unknown	5088(12.4)	199(14.35)	3464(12.59)	1247(11.90)	178(10.91)	
Adjuvant chemotherapy						
Yes	28812(70.24)	891(64.24)	19289(70.09)	7477(71.33)	1155(70.77)	<0.0001
No	9682(23.6)	409(29.49)	6578(23.90)	2320(22.13)	375(22.98)	
Unknown	2527(6.16)	87(6.27)	1652(6.00)	686(6.54)	102(6.25)	
Adjuvant hormonal						
Yes	26265(64.03)	856(61.72)	17723(64.40)	6651(63.45)	1035(63.42)	0.2424
No	12782(31.16)	463(33.38)	8501(30.89)	3300(31.48)	518(31.74)	
Unknown	1974(4.81)	68(4.90)	1295(4.71)	532(5.07)	79(4.84)	

Breast cancer subgroups categorized according to the expression of ER, PR and HER2 exhibited a significant association with BMI categories (*p* < 0.001). The ER and/or PR+/HER2- and ER and PR-/HER2- subtypes were more prevalent in the obese BMI category. However, the ER and/or PR+/HER2+ subtype was more prevalent in the underweight BMI category.

### Overall survival and breast cancer-specific survival

A total of 4468 deaths from any cause and 2824 deaths from breast cancer were observed over a median follow-up time of 92 months after diagnosis, with a maximum follow-up of 300 months.

After adjusting for poor prognostic factors, such as tumor size, axillary lymph node metastasis, histologic grade, ER, PR and HER2 expression, a U-shaped association between BMI and mortality was observed in the total cohort (Table 2). Compared with patients in the normal BMI category, those in the underweight BMI category exhibited significantly worse OS (adjusted hazard ratio [HR] 1.23, 95 % confidence interval [CI] 1.05 to 1.44, *p* = 0.0118), as did those in the obese BMI category (adjusted HR 1.29, 95 % CI 1.13 to 1.48, *p* = 0.0002). Patients in the underweight BMI category (adjusted HR 1.26, 95 % CI 1.03 to 1.54, *p* = 0.0219) and the obese BMI category (adjusted HR 1.21, 95 % CI 1.02 to 1.43, *p* = 0.0321) exhibited significantly worse BCSS compared with those in the normal BMI category.

**Table 2:** Cox’s proportional hazard regression model for overall survival (OS) and breast cancer specific survival (BCSS)

Characteristic	Overall survival						Breast cancer specific survival					
	Alive	Death	HR (95 % CI)	*p* value	Adjust HR (95 % CI)	*p* value	Alive	Death	HR (95 % CI)	*p* value	Adjust HR (95 % CI)	*p* value
No. of participants	36553	4468					98197	2824				
Age (years)												
≤ 35	3330(9.11)	407(9.11)	1.00				3479(9.11)	258(9.14)	1.00			
> 35	33223(90.89)	4061(90.89)	1.04(0.94-1.15)	0.4945			34718(90.89)	2566(90.86)	1.03(0.91-1.17)	0.6453		
Tumor size (cm)												
0 - 2	20608(56.38)	1323(29.61)	1.00		1.00		21185(55.46)	746(26.42)	1.00		1.00	
> 2	15945(43.62)	3145(70.39)	2.74(2.57-2.92)	<0.0001	1.70(1.58-1.82)	<0.0001	17012(44.54)	2078(73.58)	3.16(2.91-3.44)	<0.0001	1.77(1.61-1.93)	<0.0001
Axillary lymph node metastasis												
Negative	23731(64.92)	1474(32.99)	1.00		1.00		24439(63.98)	766(27.12)	1.00		1.00	
Positive	12822(35.08)	2994(67.01)	3.43(3.22-3.65)	<0.0001	2.95(2.75-3.16)	<0.0001	13758(36.02)	2058(72.88)	4.42(4.07-4.80)	<0.0001	3.50(3.20-3.84)	<0.0001
Operation method												
Mastectomy	19830(54.25)	3532(79.05)	1.00		1.00		21094(55.22)	2268(80.31)	1.00		1.00	
Conserving surgery	16723(45.75)	936(20.95)	0.37(0.34-0.39)	<0.0001	0.55(0.51-0.59)	<0.0001	17103(44.78)	556(19.69)	0.34(0.31-0.37)	<0.0001	0.55(0.50-0.61)	<0.0001
ER/PR expression												
ER and/or PR Positive	25647(70.16)	2519(56.38)	1.00		1.00		26629(69.71)	1537(54.43)	1.00		1.00	
ER and PR Negative	10906(29.84)	1949(43.62)	1.75(1.65-1.86)	<0.0001	1.55(1.42-1.70)	<0.0001	11568(30.29)	1287(45.57)	1.87(1.73-2.01)	<0.0001	1.56(1.40-1.74)	<0.0001
HER 2 expression												
Negative	26098(71.40)	2432(54.43)	1.00		1.00		27018(70.73)	1512(53.54)	1.00		1.00	
Positive	7040(19.26)	965(21.59)	1.42(1.32-1.53)	<0.0001	1.11(1.03-1.20)	0.0061	7402(19.38)	603(21.35)	1.41(1.29-1.55)	<0.0001	1.08(0.98-1.19)	0.1128
Unknown	3415(9.34)	1071(23.97)	2.23(2.07-2.41)	<0.0001	1.70(1.58-1.84)	<0.0001	3777(9.89)	709(25.10)	2.45(2.23-2.68)	<0.0001	1.91(1.74-2.11)	<0.0001
BMI calssification												
Underweight BMI	1229(3.36)	158(3.54)	1.16(0.98-1.36)	0.0784	1.23(1.05-1.44)	0.0118	1284(3.36)	103(3.65)	1.18(0.97-1.44)	0.0974	1.26(1.03-1.54)	0.0219
Normal BMI	24766(67.75)	2753(61.62)	1.00		1.00		25774(67.48)	1745(61.79)	1.00		1.00	
Overweight BMI	9164(25.07)	1319(29.52)	1.26(1.18-1.35)	<0.0001	1.15(1.07-1.23)	<0.0001	9651(25.27)	832(29.46)	1.25(1.15-1.36)	<0.0001	1.13(1.04-1.22)	0.0052
Obese BMI	1394(3.81)	238(5.33)	1.51(1.32-1.72)	<0.0001	1.29(1.13-1.48)	0.0002	1488(3.90)	144(5.10)	1.42(1.19-1.68)	<0.0001	1.21(1.02-1.43)	0.0321
Histologic grade												
Low (grade1 - 2)	18316(50.11)	1727(38.65)	1.00		1.00		19002(49.75)	1041(36.86)	1.00		1.00	
High (Grade 3)	13631(37.29)	2259(50.56)	1.73(1.62-1.84)	<0.0001	1.32(1.23-1.41)	<0.0001	14401(37.70)	1489(52.73)	1.86(1.72-2.02)	<0.0001	1.36(1.25-1.48)	<0.0001
Unknown	4606(12.60)	482(10.79)	1.02(0.92-1.13)	0.6849	1.04(0.94-1.15)	0.494	4794(12.55)	294(10.41)	1.04(0.92-1.19)	0.5269	1.08(0.95-1.23)	0.2561
Adjuvant chemotherapy												
Yes	25339(69.32)	3473(77.73)	1.00		1.00		26475(69.31)	2337(82.75)	1.00		1.00	
No	9002(24.63)	680(15.22)	0.58(0.53-0.63)	<0.0001	1.41(1.29-1.55)	<0.0001	9354(24.49)	328(11.61)	0.42(0.37-0.47)	<0.0001	1.12(0.99-1.28)	0.0753
Unknown	2212(6.05)	315(7.05)	1.02(0.91-1.14)	0.7874	1.09(0.97-1.24)	0.1448	2368(6.20)	159(5.63)	0.75(0.64-0.88)	0.0005	0.79(0.67-0.94)	0.0062
Adjuvant hormonal												
Yes	23785(65.07)	2480(55.51)	1.00		1.00		24743(64.78)	1522(53.90)	1.00		1.00	
No	11000(30.09)	1782(39.88)	1.53(1.44-1.63)	<0.0001	1.07(0.98-1.17)	0.1399	11604(30.38)	1178(41.71)	1.63(1.51-1.76)	<0.0001	1.12(1.01-1.25)	0.0414
Unknown	1768(4.84)	206(4.61)	1.13(0.98-1.30)	0.0920	1.02(0.88-1.19)	0.7627	1850(4.84)	124(4.39)	1.10(0.92-1.32)	0.3058	1.04(0.86-1.25)	0.6977

Abbreviations: *OS* overall survival, *BCSS* breast cancer specific survival, *HR* hazard ratio

Data presented as n (%) and HR (95 % CI)

HRs are unadjusted or adjusted based on Cox’s proportional-hazard regression models

Patient age; tumor size; histologic grade; lymph node status; operation method; adjuvant treatment; ER/PR status and HER2 expression included in the multivariate analysis model

In the ER and/or PR+/HER2- subgroup, patients in the obese BMI category exhibited significantly worse OS (adjusted HR 1.48, 95 % CI 1.18 to 1.85, *p* = 0.0006) and BCSS (adjusted HR 1.31, 95 % CI 1.13 to 1.52, *p* = 0.0003) compared with those in the normal BMI category (Table 3). However, no significant difference was observed in the OS (*p* = 0.1269) and BCSS (*p* = 0.2684) rate between patients in the normal BMI category and the underweight BMI category. Conversely, in the ER and PR-/HER2+ subgroup, patients in the underweight BMI category exhibited significantly worse OS (adjusted HR 1.67, 95 % CI 1.12 to 2.47, *p* = 0.0113) and BCSS (adjusted HR 1.79, 95 % CI 1.11 to 2.90, *p* = 0.0179) compared with those in the normal BMI category. However, obese BMI was not associated with decreased OS (*p* = 0.4247) or BCSS (*p* = 0.5683) in the ER and PR-/HER2+ subgroup. In the ER and/or PR+/HER2+ and ER and PR-/HER2- subgroups, BMI categories did not exhibit a significant association with OS and BCSS. In the unknown subgroup, patients in the obese BMI category exhibited significantly worse OS (adjusted HR 1.36, 95 % CI 1.04 to 1.79, *p* = 0.0261) but not worse BCSS (adjusted HR 1.36, 95 % CI 0.97 to 1.90, *p* = 0.0747) compared with those in the normal BMI category.

**Table 3:** Cox’s proportional hazard regression model for overall survival (OS) and breast cancer specific survival (BCSS) among breast cancer subtypes

Subtype	Overall survival						Breast cancer specific survival					
	Alive	Death	HR (95 % CI)	*p* value	Adjust HR (95 % CI)	*p* value	Alive	Death	HR (95 % CI)	*p* value	Adjust HR (95 % CI)	*p* value
ER and/or PR + and HER 2 -												
No. of participants	19674	1420					20248	846				
Underweight BMI	659(3.35)	47(3.31)	1.17(0.87-1.57)	0.2902	1.26(0.94-1.69)	0.1269	678(3.35)	28(3.31)	1.17(0.80-1.71)	0.4239	1.24(0.85-1.82)	0.2684
Normal BMI	13352(67.87)	827(58.24)	1.00		1.00		13686(67.59)	493(58.27)	1.00		1.00	
Overweight BMI	4901(24.91)	462(32.54)	1.48(1.32-1.66)	<0.0001	1.32(1.18-1.48)	<0.0001	5084(25.11)	279(32.98)	1.49(1.29-1.73)	0.0035	1.30(0.96-1.76)	0.0874
Obese BMI	762(3.87)	84(5.92)	1.74(1.39-2.18)	<0.0001	1.48(1.18-1.85)	0.0006	800(3.95)	46(5.44)	1.57(1.16-2.12)	<0.0001	1.31(1.13-1.52)	0.0003
ER and/or PR + and HER 2 +												
No. of participants	3691	427					3851	267				
Underweight BMI	130(3.52)	14(3.28)	0.95(0.56-1.62)	0.8463	1.07(0.63-1.83)	0.802	139(3.61)	5(1.87)	0.54(0.22-1.31)	0.1737	0.61(0.25-1.48)	0.2705
Normal BMI	2612(70.77)	297(69.56)	1.00		1.00		2726(70.79)	183(68.54)	1.00		1.00	
Overweight BMI	827(22.41)	102(23.89)	1.06(0.85-1.33)	0.5960	1.02(0.81-1.28)	0.875	856(22.23)	73(27.34)	1.24(0.95-1.63)	0.1177	1.18(0.90-1.55)	0.2251
Obese BMI	122(3.31)	14(3.28)	1.05(0.61-1.79)	0.8608	0.94(0.55-1.61)	0.8193	130(3.38)	6(2.25)	0.72(0.32-1.62)	0.4277	0.62(0.27-1.39)	0.2449
ER and PR - and HER 2 +												
No. of participants	3349	538					3551	336				
Underweight BMI	94(2.81)	27(5.02)	1.79(1.21-2.65)	0.0034	1.67(1.12-2.47)	0.0113	103(2.90)	18(5.36)	1.88(1.16-3.05)	0.0099	1.79(1.11-2.90)	0.0179
Normal BMI	2288(68.32)	344(63.94)	1.00		1.00		2418(68.09)	214(63.69)	1.00		1.00	
Overweight BMI	847(25.29)	141(26.21)	1.09(0.90-1.33)	0.3926	0.94(0.77-1.14)	0.5093	900(25.34)	88(26.19)	1.09(0.85-1.40)	0.4921	0.93(0.73-1.19)	0.5715
Obese BMI	120(3.58)	26(4.83)	1.38(0.93-2.06)	0.1132	1.18(0.79-1.76)	0.4247	130(3.66)	16(4.76)	1.35(0.81-2.24)	0.2496	1.16(0.70-1.93)	0.5683
ER and PR - and HER 2 -												
No. of participants	6424	1012					6770	666				
Underweight BMI	215(3.35)	34(3.36)	1.09(0.77-1.54)	0.6222	1.19(0.84-1.68)	0.3333	225(3.32)	24(3.60)	1.14(0.76-1.72)	0.5347	1.27(0.84-1.92)	0.2606
Normal BMI	4206(65.47)	610(60.28)	1.00		1.00		4402(65.02)	414(62.16)	1.00		1.00	
Overweight BMI	1727(26.88)	311(30.73)	1.20(1.05-1.38)	0.0080	1.07(0.93-1.23)	0.3301	1849(27.31)	189(28.38)	1.07(0.90-1.27)	0.4398	0.93(0.79-1.11)	0.4320
Obese BMI	276(4.30)	57(5.63)	1.37(1.04-1.80)	0.0234	1.18(0.90-1.55)	0.2395	294(4.34)	39(5.86)	1.38(0.99-1.91)	0.0571	1.16(0.84-1.61)	0.3749
Unknown												
No. of participants	3415	1071					3777	709				
Underweight BMI	131(3.84)	36(3.36)	0.98(0.70-1.37)	0.8923	1.11(0.79-1.55)	0.5528	139(3.68)	28(3.95)	1.16(0.79-1.70)	0.44	1.29(0.87-1.89)	0.2010
Normal BMI	2308(67.58)	675(63.03)	1.00		1.00		2542(67.30)	441(62.20)	1.00		1.00	
Overweight BMI	862(25.24)	303(28.29)	1.18(1.03-1.35)	0.0153	1.12(0.98-1.28)	0.1042	962(25.47)	203(28.63)	1.20(1.02-1.42)	0.0283	1.15(0.97-1.36)	0.1030
Obese BMI	114(3.34)	57(5.32)	1.61(1.23-2.11)	0.0005	1.36(1.04-1.79)	0.0261	134(3.55)	37(5.22)	1.56(1.11-2.18)	0.0099	1.36(0.97-1.90)	0.0747

Abbreviations: *OS* overall survival, *BCSS* breast cancer specific survival, *HR* hazard ratio

Data presented as n (%) and HR (95 % CI)

HRs are unadjusted or adjusted based on Cox’s proportional-hazard regression models

Patient age; tumor size; histologic grade; lymph node status; operation method; adjuvant treatment; ER/PR status and HER2 expression included in the multivariate analysis model

## Discussion

In our total cohort analysis, underweight and obese breast cancer patients exhibited significantly poorer OS and BCSS compared with normal BMI category breast cancer patients, suggesting a U-shaped relationship, as has been previously suggested [7, 13, 14, 17]. This is the largest study to suggest that breast cancer patients with a normal BMI range at diagnosis exhibit the most favorable breast cancer outcomes.

Similar to our finding, a previous study using the KBCR database demonstrated that underweight BMI is an independent negative prognostic factor for OS and BCSS after adjustment. However, in a previous study, neither obese patients nor overweight patients exhibited significant differences in OS and BCSS compared with normal-weight patients [18]. Two studies using the KBCR database reported different results for the prognostic significance of obesity in breast cancer patients. A potential explanation for the differing results is that more recent breast cancer patients (between 2007 and 2008) were included our study. Because an increase in overweight BMI and obesity has been noted in South Korean adults [33], our study, which included more recent breast cancer patients, may provide a more accurate analysis of the prognostic significance of obesity in Korean breast cancer patients.

This study is the first to further explore results with respect to both BMI at diagnosis and the four breast cancer subtypes, enabling better identification of women at highest risk of poor outcomes.

In the ER and/or PR+/HER2- subgroup, obese breast cancer patients exhibited significantly worse OS and BCSS compared with normal and underweight BMI breast cancer patients. Previous studies have demonstrated that obesity is associated with an increase in OS or BCSS in patients with ER and/or PR positive breast cancer but is not in patients with ER and PR negative breast cancer [1, 4, 10, 11, 14]. Several hypotheses may explain why obese patients with HR-positive breast cancer exhibit worse survival [8, 34–36]. Obesity is related to the increased peripheral conversion of androgenic precursors to estradiol due to increased aromatase enzyme activity from large amounts of adipose tissue and is also related to decreased sex hormone-binding globulin [8, 34]. Additionally, obesity can increase insulin and insulin-like growth factors and obesity-related regulatory proteins, such as leptin and adiponectin [35, 36]. As a result, high circulating bioavailable estrogen, growth factors and regulatory proteins could have a carcinogenic effect, promoting tumor growth and progression, in breast cancer cells expressing the estrogen receptor.

In contrast to the ER and/or PR+/HER2- subgroup, underweight breast cancer patients exhibit significantly worse OS and BCSS compared with normal and obese BMI category breast cancer patients in the ER and PR-/HER2+ subgroup. Recently, two studies evaluated the correlation between BMI and disease-free survival in HER2-positive breast cancer patients [11, 37]. One study reported that obesity decreases survival compared with normal weight [11], but the second study reported conflicting results [37]. Because these studies were analyzed only in the context of obese versus non-obese HER2-positive patients, including ER and/or PR positive and ER and PR negative breast cancer patients in the small sample size, these studies have not demonstrated whether an underweight BMI is associated with an increased risk of mortality relative to normal weight in the ER and PR-/HER2+ subgroup. The relationship between underweight BMI and decreased survival might be at least partly explained by the presence of circulating tumor cells (CTCs) in the peripheral blood of breast cancer patients. CTCs that have detached from the primary tumor site may reach a secondary organ and lead to metastases [38]. Furthermore, alterations of the circulating immune cells may influence tumor progression and the efficacy of systemic antitumor treatments. Chronic undernutrition and micronutrient deficiency compromise the cytokine response and affect immune cell trafficking, which might affect the tumor-immune system interaction in other organs [39].

In the ER and/or PR+/HER2+ and ER and PR-/HER2- subgroups, BMI categories did not exhibit a significant association with OS and BCSS. Similar to our finding, one study reported that obesity was not associated with decreased survival in patients with triple-negative breast cancer [40]. Because the impact on BMI and breast cancer outcomes was masked by the effect of the ER and/or PR+/HER2- and ER and PR-/HER2+ subgroups, a weaker association between BMI and poor outcomes may exist in patients in the ER and/or PR+/HER2+ and ER and PR-/HER2- subgroup.

Our study has several strengths and limitations. The main strength of our study is its inclusion of a large sample (4468 deaths from any cause, 2824 deaths from breast cancer among 41,021 breast cancer patients), permitting a detailed examination across multiple BMI categories during the long follow-up period. Furthermore, our study is the first to investigate the prognostic significance of BMI in four different breast cancer subtypes. However, our study was limited by the information available in the KBCR database. First, registered patients in the KBCR database were heterogeneous with respect to breast cancer stage, IHC staining results, and presence of comorbidities. Additionally, the essential registry data (BMI and HR expression) were only available for 62.84 % of invasive breast cancer patients in the KBCR database. Therefore, the possibility of selection bias remains. Second, although total sample size is larger compared with previous studies, the sample size of underweight and obese BMI subjects is small to draw a conclusion on the independent effect of BMI. Finally, the ethnic homogeneity of the KBCR database may limit the generalizability of our finding to other racial and ethnic groups.

## Conclusions

In conclusion, our results indicated a U-shaped relationship between BMI at diagnosis and poor OS and BCSS among all breast cancer patients, with the lowest risk observed among breast cancer patients with normal BMIs (18.5 - 24.9 kg/m^2^). Among breast cancer patients with ER and/or PR+/HER2- tumors, obese individuals exhibit significantly poorer OS and BCSS, whereas among those with ER and PR-/HER2+ tumors, underweight patients exhibit significantly poorer OS and BCSS compared with breast cancer patients with normal BMIs. Although obesity and underweight BMI at diagnosis are poor prognostic factors in pooled breast cancer samples, BMI at diagnosis exhibited a different impact on breast cancer prognosis in specific breast cancer subtypes. Therefore, BMI at diagnosis and breast cancer subtype should be considered simultaneously in various treatment decision processes and surveillance schedules.

## Abbreviations

HR: Hormone receptor
HER2: Human epidermal growth factor receptor 2
OS: Overall survival
BCSS: Breast cancer-specific survival
ER: Estrogen receptor
PR: Progesterone receptor
IHC: Immunohistochemistry
FISH: Fluorescence in situ hybridization
HR: Hazard ratio
CI: Confidence interval
